# Effect of incorporation of soy flour to wheat flour on nutritional and sensory quality of biscuits fortified with mushroom

**DOI:** 10.1002/fsn3.228

**Published:** 2015-03-28

**Authors:** Tasnim Farzana, Suman Mohajan

**Affiliations:** Quality Control Research Section, Institute of Food Science and Technology (IFST), Bangladesh Council of Scientific and Industrial Research (BCSIR)Dhaka, Bangladesh

**Keywords:** Biscuits, food fortification, mushroom, protein, soy flour, wheat flour

## Abstract

The research study was conducted to evaluate the quality characteristics of soy-mushroom-enriched biscuits which could be used as a protein supplemented cereal snack food. In this study, wheat flour was replaced with soy flour at different levels that is 20% (T3), 15% (T2), and 10% (T1) and without soy flour was kept as control (T_o_). Mushroom was added in both biscuits. Biscuits were analyzed for chemical and sensory parameters. Protein content of soy flour-supplemented biscuits increased from 11.07% to 17.86% as compared to control along with a significant increased in fat (17.36–20.89%), fiber (0.48–0.92%), iron (1.56–1.99 mg/100 g), and energy value (463–485 Kcal/g). Ash content also increased but not significantly. Results from chemical analyses and organoleptic evaluation indicate that good quality biscuits can be prepared by substituting wheat flour with 15% soy flour and addition of mushroom powders may affect the backing quality. Protein Energy Malnutrition (PEM) of the Bangladeshi population can be reduced through the development of biscuits in this way.

## Introduction

Protein Malnutrition is widely recognized as a major health problem in Bangladesh due to cereal-based dietary pattern. The protein quality of the cereal-based diet can be improved by fortifications. According to FAO standards (FAO 1995), suggestion, to meet the recommended dietary allowances of infants, preschool children, adolescent girls, pregnant and lactating women, low-cost supplementary foods could be processed domestically by simple, inexpensive processing technology. The use of protein-calorie sources of vegetables or other origins as a supplementation on regular diet has been proposed a possible solution to this problem.

Nowadays, bakery food products, especially biscuits are becoming very popular in Bangladesh in rural as well as urban areas among all the age groups due to its several attractive features, including wider consumption, low cost among other processed foods, varied taste, easy availability and good eating quality, and relatively long shelf life (Gandhi et al. 2001; Ayo and Olawale 2003). Biscuits are high in carbohydrates, fat, and calorie but low in fiber, vitamin, and mineral which make it unhealthy for daily use (Serrem et al. 2011). Moreover, biscuits have only about 6–7% protein (Agarwal 1990). This may be achieved through incorporation of protein-rich ingredients from soybean and mushroom as a fortification of biscuits.

Soybean (Glycine max), a species of legume, a miracle bean, is an excellent health food and it contains 43.2% good quality protein but only minimal saturated fat, 21% carbohydrates (Gopalan et al. 1999), and sufficient amounts of minerals and vitamins. Moreover, most of the oilseeds contain 40–50% oil, where as soybean contains about 18% of oil (American Soybean Association 2004). Amino acid profile of soy protein is excellent amongst plant proteins. Hence, it is superior to other plant proteins as it contains most of the essential amino acids except methionine (FAO 1970), which is abundant in cereals, and it is the most economical source of dietary protein. Soy protein directly lowers serum cholesterol levels (Mirrahimi et al. 2010). Soybeans also contain biologically active or metabolic proteins such as enzymes, trypsin inhibitors, hemagglutinins, and cysteine proteases very similar to papain (American Soybean Association 2004). The soy cotyledon storage proteins are important for human nutrition. Soybean contains isoflavones, which are said to have potential anticancer effects. It contains two primary isoflavones called Genistein and Diadzein and a minor one called as Glycitein. They retard bone loss in premenstrual and postmenstrual women, soluble fiber in soy foods control blood sugar. Soy foods are quite important to us as they reduce the risk of heart disease. Regular consumption of soy food delays the process of aging and also improves mental and physical abilities, memory power, and hemoglobin levels of children (American Soybean Association 2004). Owing to these qualities, soybean has long been used in supplementary foods.

Mushroom, another miracle food, is now being used in supplementary food. Mushrooms have a great potential due its high and good quality proteins (20–40% on dry weight basis), Vitamins (Vitamin B- complex), and minerals (Singh et al. 1995). So mushrooms can be dried and converted into powdered form, which can be used for fortification in baked products like bread, biscuits, etc. In view of this consideration, the present work was designed to economically complement and fortify wheat flour with soy flour and mushroom for biscuit production and to study the effect of different combination of soy flour on the nutritional and sensory quality of the developed biscuits.

## Materials and Methods

The study was carried out in the laboratory of Quality Control Research Section of Institute of Food Science and Technology (IFST), Bangladesh Council of Scientific and Industrial Research (BCSIR), Bangladesh.

### Raw materials

Soybean was collected from the Bangladesh Agricultural Research Institute. Oyster mushroom (*Pleurotus ostreatus*) was collected from the National Mushroom Development and Extension Center, Savar, Bangladesh. Other ingredients were collected from the local market.

### Preparation of raw materials

Soybean seeds were sorted and soaked in water for 12 h. Thereafter, the seed coat was removed and drained. The seeds were then cleaned and boiled for 30 min and dried at 65°C for 9 h. The dried seeds were milled into flour. The flours were screened through a 0.25 mm sieve and stored at 4°C in a refrigerator to prevent spoilage particularly rancidity until usage. The process ensures effective removal of most antinutritional factors.

Mushrooms were dried in a thermostatically controlled oven with an air fan to 50°C for 6 h and ground into powder in a grinder. The flour obtained was sieved using 0.25 mm sieve.

### Method of preparation of biscuit

**Figure 1:**
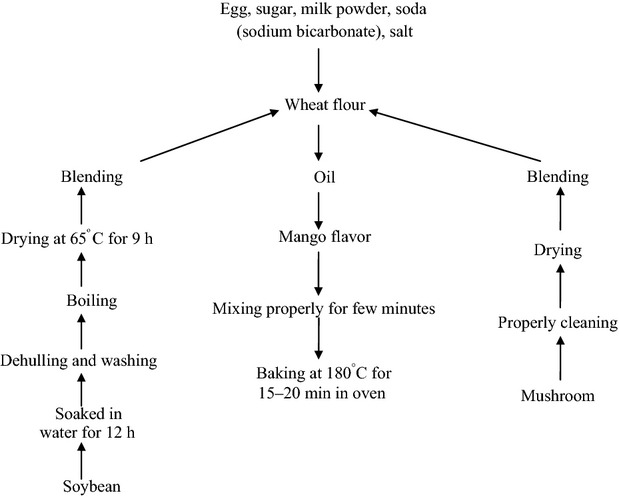
Flow chart for the preparation of soya-mushroom biscuit.

Wheat flour was replaced with soy flour in three proportions 10, 15, and 20% and designed as T1, T2, and T3, respectively, whereas T_o_ (without soy flour) was kept as control. Fixed amount of mushroom (5%) was added in all the treatments and control. The formulation and preparation of biscuits is shown in Table1 and Figure1, respectively.

**Table 1:** Formulation of the biscuits

Sample table of formulation of the product									
Samples	Wheat flour (%)	Soy flour (%)	Mushroom (%)	Milk powder (%)	Egg (mL)	Sugar (g)	Sodium bicarbonate (g)	Salt (NaCl) (g)	Oil (mL)
Control	85	0	5	10	24.0	40.0	1.0	0.6	20.0
T1	75	10	5	10	24.0	40.0	1.0	0.6	20.0
T2	70	15	5	10	24.0	40.0	1.0	0.6	20.0
T3	65	20	5	10	24.0	40.0	1.0	0.6	20.0

### Sensory analysis

The organoleptic test of the products was done by the 9-point hedonic scale scorecard, especially prepared for the purpose. A 10-member trained panelist was selected from the staff members of the Institute of Food Science and Technology (IFST), Bangladesh Council of Scientific and Industrial Research (BCSIR), Bangladesh. Each attribute was scored based on its intensity scaled on a 9-point hedonic scale (1 = disliked extremely, 2 =  disliked very much, 3 = disliked moderately, 4 =  disliked slightly, 5 = neither liked or disliked, 6 = liked slightly, 7 = like moderately, 8 = liked very much, 9 = liked very extremely) for color, flavor, texture, and taste.

### Method of analysis

#### Proximate analysis

The proximate composition (i.e., moisture, protein, fat, fiber, carbohydrate) and total energy of soya-mushroom biscuits were determined according to the standard analytical methods (AOAC 2000).

#### Determination of moisture

Moisture content was determined by drying a sample in an oven at 100°C for 12 h, the weight loss incurred was calculated as: 

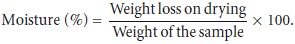


#### Determination of crude protein

Crude protein content of the samples was determined using the Kjeldahl method. The method consists of three basic steps: (1) digestion of the sample in sulfuric acid with a catalyst, which results in conversion of nitrogen to ammonia; (2) distillation of the ammonia into a trapping solution; and (3) quantification of the ammonia by titration with a standard solution.

According to this method, percentage of crude protein content of the samples = % nitrogen × 6.25.

#### Determination of total ash

Crucibles were first dried for about 2 h at 100°C in an oven and placed in a desiccator. They were cooled and about 2.0 g of sample was weighed into the crucible. The samples were then placed in a furnace at 600°C for 4 h. Percentage ash content was determined by weighing the resulting inorganic residue. 

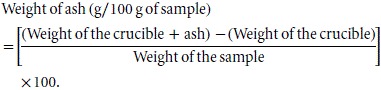


#### Determination of fat

Fat content was determined using the Soxhlet extraction method. In this method, fat was determined by extracting the dried materials (food samples) with a light petroleum fraction in a continuous extraction apparatus. The solvent was distilled off and the extract was dried and weighed.

#### Determination of crude fiber

The moisture and fat-free sample was boiled with 0.255N H_2_SO4 and 0.313N NaOH, consecutively, for 30 min under a reflux condenser and each time the sample was washed well with boiling water to remove acid and alkali residues. The sample was then transferred into a crucible, dried overnight at 100°C and weighed (W_1_) in an analytical balance. The crucible was heated in a muffle furnace at 600°C for 20 min, cooled, and weighed again (W_2_). The difference in the weights (W_1_ – W_2_) represents the weight of crude fiber. 

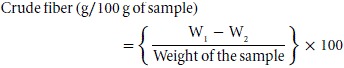


#### Determination of total carbohydrate

The content of the available carbohydrate was determined by the following equation: 

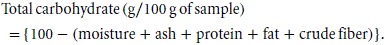


#### Determination of Iron content

Estimation of Iron by WONG's Method (Wong 1928).

#### Determination of energy content

Metabolizable energy was calculated following the formula below: 

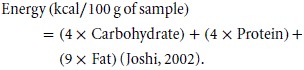


### Statistical analysis

Data analysis was performed using Statistical Package for the Social Sciences (SPSS version 15.0 SPSS Inc. Chicago, Illinois, U.S.A). Values were expressed as percentage and mean ± SD. One way ANOVA was used for determining the significance/nonsignificance of results. Means were separated using *t*-test.

## Results and Discussion

### Proximate composition of soy flour

The nutritional compositions of dehulled soy flour were moisture 1.4%, protein 49.3%, fat 24.9%, ash 2.8%, fiber 3.0%, and total carbohydrate 18.6% in dry weight. The protein and fat content is slightly higher than the results of Gopalan et al. (1999) on a dry basis.

### Proximate composition of oyster mushroom

The nutritional compositions of oyster mushroom (*Pleurotus ostreatus*) powder were moisture 4.0%, protein 31.8%, fat 2.5%, ash 7.0%, fiber 12.5% and total carbohydrate 42.2% in dry weight. The protein, ash, fiber, and fat content were almost similar to the study of Michael et al. (2011).

### Chemical composition of wheat-soy-mushroom biscuits

Chemical composition of our developed biscuits with varying soy flour percentage keeping mushroom and other ingredients constant was given below (on a dry basis).

### Moisture and ash content

In the present study, the moisture content (3.97%) was found to be the highest in control (To) biscuit. The results showed that the moisture content gradually decreased from 3.97 to 2.19% with the increase in soy flour (Table2) are in agreement with the study of Banureka and Mahendran (2009). This may be explained as soy flour contained a greater amount of total dry solid with high emulsifying properties compared to wheat flour. Due to low moisture content of the beans, moisture content of the biscuit decreased with the increasing amount of soy flour in the blend. This is supported by the findings of Sutharshan et al. (2001) who reported that increase in proportion of soy flour reduces the moisture content of the soy bean flour supplemented biscuits.

**Table 2:** Proximate analysis soy-mushroom biscuits

Parameters	To	T1	T2	T3
Moisture (%)	3.97 ± 0.06	3.08 ± 0.05^a^	2.58 ± 0.04^ab^	2.19 ± 0.05^acd^
Ash (%)	1.50 ± 0.05	1.59 ± 0.04	1.68 ± 0.06	1.75 ± 0.09
Protein (%)	11.07 ± 0.06	13.46 ± 0.08^a^	14.87 ± 0.07^ab^	17.86 ± 0.09^acd^
Fat (%)	17.36 ± 0.08	18.57 ± 0.07^a^	18.97 ± 0.07^ab^	20.89 ± 0.03^acd^
Fiber (%)	0.48 ± 0.04	0.64 ± 0.03^a^	0.76 ± 0.05^ab^	0.92 ± 0.04^acd^
Iron (mg/100 g)	1.56 ± 0.02	1.68 ± 0.03^a^	1.83 ± 0.05^ab^	1.99 ± 0.04^acd^
Carbohydrate (%)	65.62 ± 0.29	62.66 ± 0.27^a^	61.14 ± 0.29^ab^	56.38 ± 0.30^acd^
Energy (Kcal/g)	463.00 ± 0.20	471.61 ± 0.13^a^	474.77 ± 0.25^ab^	485.00 ± 0.57^acd^

Values are means of triplicates ± standard deviation.

Values with the same superscript in a column are not significantly different (*P* > 0.05).

KEYS: T1 = 10% soy flour; T2 = 15% soy flour; T3 = 20% soy flour.

The highest ash content (1.75%) was found in treatments T3 (20%) and lowest value (1.50%) was recorded in control (To) biscuit. In treatments T2 (15%) and T1 (10%), the ash content was found to be 1.68% and 1.59%, respectively The result showed that the ash content gradually increased from 1.50% to 1.76% with an increase in the percentage of soy flour as shown in Table2, opposed by a previous study conducted by Akubor and Ukwuru (2005) but in agreement with the findings of Siddiqui et al. (2003) and Ayo et al. (2014) on the supplementation of soy flour for the preparation of biscuits.

### Protein content

In the present study, the protein content was found to increase from 11.07% to 17.86%. The highest protein content (17.86%) was found in treatment T3 (20%) and lowest (11.07%) was recorded for control (To) biscuit. In treatment groups T2 (15%) and T1 (10%), protein content was found to be 14.87% and 13.46%, respectively (Table2). This trend of increase in protein content in the treatments as compared to control was supported by several studies (Siddiqui et al. 2003; Banureka and Mahendran 2009; Ayo et al. 2014). This increase could be due to the increase in the proportion of soy flour in the flour blend as soybean is a high-protein legume and an excellent complement to lysine-limited cereal protein and incorporation of soy flour inevitable increases the protein content in the biscuits. Hence, the basis for the use of soy flour as an economical protein supplement in biscuit, bread, pasta, and other cereal products (Hegstad 2008).

One important point in this study is that increased value of protein was found not only in the treatment group but also in control as compared to other previous studies (Siddiqui et al. 2003; Banureka and Mahendran 2009; Awasthi et al. 2012; Ayo et al. 2014) where the value of protein was observed lower in their experiments. This could be due to incorporation of mushroom in the flour blend. Mushroom is a good source of high-quality protein (20–40% on dry weight basis), vitamins (vitamin B- complex), and minerals (Singh et al. 1995). So mushrooms can be dried and converted into the powdered form, which can be used for fortification in baked products like bread, biscuits, etc. It could be assumed that addition of soy flour and mushroom in biscuit has a greater potential in overcoming protein-calorie malnutrition in the world (Akubor and Ukwuru 2005).

### Fat content

The fat content of the biscuits increased from 17.36% to 20.89% with the increase in soy flour. The highest fat content (20.89%) was found in treatment T3 (20%) and lowest (17.36%) was recorded for control (To) biscuit. In treatments T2 (15%) and T1 (10%), the fat content was found to be 18.97% and 18.57%, respectively (Table2). This trend of increase is in agreement with previous studies (Akubor and Ukwuru 2005; Banureka and Mahendran 2009; Ayo et al. 2014), but opposite of the findings of Siddiqui et al. (2003) on the supplementation of soy flour for the preparation of biscuits. The increase in fat content in the present study may be explained as soy flour is globally considered as the number one edible oil source, containing a higher percentage of fat than wheat flour. Reddy (2004) reported that soy flour contained 20–24% of fat, whereas wheat flour contains 0.9–1.1% and most of which are unsaturated in nature. The increased fat content in the study could be due to the increase in the proportion of soy flour in the flour blend. Soybean oil is 61% polyunsaturated fat and 24% monounsaturated fat which is comparable to the total unsaturated fat content of other vegetable oils (85%). The two polyunsaturated fats, including the two essential fatty acids, linoleic, and linolenic, that are not produced in the body, aid the body's absorption of vital nutrients and are required for human health (Hegstad 2008).

### Fiber content

The fiber content of the biscuit increased from 0.48% to 0.92% with the increase in supplementation of soy flour. The highest fiber content (0.92%) was found in treatment T3 (20%) and lowest (0.48%) was recorded for control (To) biscuit. In treatments T2 (15%) and T1 (10%), the fiber content was found to be 0.76% and 0.64%, respectively (Table2). Similar trends in increase in fiber content were also reported by Ayo et al. (2014) on the supplementation of malted soy flour on the production of biscuits. The increase in fiber content could be due to the increase in soy flour in the blended flour as supported by a study of Ndife et al. (2011) on soy flour supplementation in the production of bread. According to well-documented studies, it is now accepted that dietary fiber plays a significant role in the prevention of several diseases such as; cardiovascular diseases, diverticulosis, constipation, irritable colon, cancer, and diabetes (Slavin 2005; Elleuch et al. 2011). So, this soya-mushroom enriched biscuit may be helpful in preventing these cases.

### Carbohydrate content

Carbohydrate content was gradually decreased with the increase in supplementation of soy flour. Highest carbohydrate content was observed in control (To) (65.62%) and lowest was reported in treatment T3 (56.38%) (Table2). Similar trend in decrease in carbohydrate content was also reported by Ayo et al. (2014) on the supplementation of malted soy flour on the production of biscuits. The decrease in carbohydrate content could be due to the low carbohydrate content of added soy flour (21%) (Gopalan et al. 1999).

### Iron content

The Iron content of the biscuit increased from 1.56% to 1.99% with the increase in supplementation of soy flour. The highest Iron content (1.99%) was found in treatment T3 (20%) and lowest (1.56%) was recorded for control (To) biscuit. In treatment T2 (15%) and T1 (10%), iron content was found as 1.83% and 1.68%, respectively (Table2). This increase in iron content may be increased in soy flour in the mixture. It was reported by several studies that mineral content of wheat flour was increased by the supplementation of soy flour. Rawat et al. (1994) stated that soy supplemented chapattis have a higher level of Fe than prepared from whole wheat flour. Maqbool et al. (1987) also reported that Fe content of wheat *rotis* was increased with supplementation, which is in line with the present study.

### Energy value

Food energy is the amount of caloric value available from food through oxidation. The greatest amount of energy (9 kcal/g) is obtained from oxidation of fats while proteins and most carbohydrates have about 4 kcal/g. In the present study, the calorie content of the biscuits has been increased from 463.00 to 484.97 kcal with the addition of soy flour (Table2). A similar trend was also reported by Banureka and Mahendran (2009).

### Sensory evaluation soy-mushroom biscuits

In the present study, sensory scores of biscuit enriched with 10% (T1), 15% (T2), and 20% (T3) soy flour keeping the mushroom content fixed (5%), showed that with regard to flavor, taste, body texture, color and appearance, and overall acceptability, the sensory characteristics of T1 (10%) were found to be the best and T2 (15%) was closer T1 (10%). Other treatments T3 (20%) and control (To) were also found acceptable (Table3).

**Table 3:** Sensory attributes of soy-mushroom biscuits incorporated with different levels of soy flour

Treatments	Taste	Color	Texture	Flavour	Overall acceptability
Control (T_0_)	8.5 ± 0.2	7.9 ± 0.2	7.8 ± 0.2	7.9 ± 0.3	8.0 ± 0.2
T_1_	8.2 ± 0.3	8.6 ± 0.3	7.6 ± 0.3	7.7 ± 0.3	8.4 ± 0.2
T_2_	8.0 ± 0.2	8.2 ± 0.2	7.5 ± 0.2	7.6 ± 0.2	8.2 ± 0.3
T_3_	7.2 ± 0.3^acd^	7.6 ± 0.3^d^	6.9 ± 0.3^ad^	7.2 ± 0.2	7.2 ± 0.3^acd^

Values are means of triplicates ± standard deviation.

Values with the same superscript in a column are not significantly different (*P* > 0.05).

KEYS: T1 = 10% soy flour; T2 = 15% soy flour; T3 = 20% soy flour.

The taste is the primary factor which determines the acceptability of any product, which has the highest impact as far as market success of product, is concerned. The score for taste had been decreased from 8.5 to 7.2 with the increase in the level of substitution of soy flour. Biscuit containing 20% soy flour (T3) was rated poorest in taste (7.2). The control (To) has the highest mean score (8.5). The mean scores for color of the biscuits change from 7.6 to 8.6. The highest score (8.6) was obtained for treatment T1. In treatment T2, the mean score for color was 8.2. On the other hand, for control (To) it was 7.9. As increase in soy flour, mean score for color was also decreasing. A similar trend was also reported by Banureka and Mahendran (2009).

The texture of the crust was related to the external appearance of the biscuit top which implies smoothness or roughness of the crust. With the increase in substitution of soy flour to the biscuits, the texture of crust was decreased from 7.8 to 6.9. The control (To) had the highest mean value and 20% soy flour (T3) added biscuit had the least mean value. In the case of flavor of the biscuit, it was decreased from 7.9 to 7.2 with an increase in the substitution of soy flour. This could be due to the beany flavour of soy flour (Akubor and Ukwuru 2005). Overall acceptability includes many implications, which is an important parameter in organo-leptic estimation. Treatment T1 that is 10% soy flour-added biscuits had the highest mean value (8.4) and T3 that is 20% soy flour-added biscuits had the least mean value (7.3) for the overall acceptability. The overall acceptability for T2 (15%) has a mean score of 8.2 very close to T1 (10%). At the 10% (T1) and 15% (T2) level of soy flour incorporation, the biscuits had higher scores for all the sensory attributes evaluated. Above this level, biscuits received a lower sensory score.

## Conclusion

This study has demonstrated that Biscuits with soy flour substitution, up to 20% were nutritionally superior to that of the whole wheat flour biscuits. Although 10% and 15% soy flour supplemented biscuit is nutritionally different, organoleptically they are close to each other. Incorporation of mushroom in the biscuit will also enhance nutritional quality. Considering biochemical analyses and organoleptic evaluation, 15% soy flour-supplemented biscuit is found to be the best. The findings of the present study may help in developing commercial processing technology for effective utilization of soy flour and mushroom powder especially in the manufacturing of biscuits. The Protein Energy Malnutrition (PEM) of the Bangladeshi population can therefore be reduced through the development of these biscuits..

## Conflict of Interest

None declared.
